# Oral Anticoagulant Therapy in Patients Receiving Haemodialysis: Is It Time to Abandon It?

**DOI:** 10.1155/2013/170576

**Published:** 2013-11-27

**Authors:** Marek Saracyn, Dorota Brodowska-Kania, Stanisław Niemczyk

**Affiliations:** Department of Internal Diseases, Nephrology and Dialysis, Military Institute of Medicine, Szaserów Street 128, 04-141 Warsaw, Poland

## Abstract

Oral anticoagulant (OAC) therapy in haemodialysis patients causes a great deal of controversy. This is because a number of pro- and anticoagulant factors play an important role in end-stage renal failure due to the nature of the disease itself. In these conditions, the pharmacokinetic and pharmacodynamic properties of the OACs used change as well. In the case of the treatment of venous thromboembolism, the only remaining option is OAC treatment according to regimens used for the general population. Prevention of HD vascular access thrombosis with the use of OACs is not very effective and can be dangerous. However, OAC treatment in patients with atrial fibrillation in dialysis population may be associated with an increase in the incidence of stroke and mortality. Doubts should be dispelled by prospective, randomised studies; at the moment, there is no justification for routine use of OACs in the above-mentioned indications. In selected cases of OAC therapy in this group of patients, it is absolutely necessary to control and monitor the applied treatment thoroughly. Indications for the use of OACs in patients with end-stage renal disease, including haemodialysis patients, should be currently limited.

## 1. Introduction

Chronic kidney disease (CKD) constitutes an increasingly serious challenge for modern medicine, both in its strictly clinical aspect and in the epidemiological one. According to different but essentially consistent estimates, it is assumed that its various stages currently affect more than 600 million people worldwide, including 10 million patients with its end stage, and 2 million patients undergoing various forms of renal replacement therapy. The high incidence and morbidity in the terminal stages of CKD are also associated with a high mortality rate, which is almost 19% of all patients undergoing different forms of dialysis treatment [1]. The most common causes of death in this population of patients include cardiovascular diseases (CVDs) (39%), infections (12%), stroke (10.3%), and neoplastic diseases (10%) [2]. The high epidemiological indices result from both the aging of the population and other concomitant diseases (such as cardiovascular diseases, diabetes, and arterial hypertension) increasingly and commonly occurring also in this group of patients. Epidemiological data fully justify the statement that CKD has become a serious social issue and, like the above-mentioned conditions, another lifestyle disease.

As the incidence and prevalence of CKD, and its end stages in particular, increase, the number of patients undergoing various forms of renal replacement therapy also constantly increases. Of the three basic treatment methods, haemodialysis (HD) therapy is the one that is most commonly applied. This is because, according to global data, more than 68% of patients requiring renal replacement therapy undergo haemodialysis; patients after renal transplant account for approximately 23% of the discussed population, while patients treated with peritoneal dialysis constitute less than 9% [3]. Prognoses for the next few years suggest a further increase in the number of patients requiring different forms of renal replacement therapy, including patients receiving haemodialysis, especially among patients with diabetes, arterial hypertension as well as the elderly ones [4].

In recent years, the indications for treatment with oral anticoagulants (OACs) as well as their use have increased significantly [5]. This phenomenon included both the entire population of patients with CKD and patients receiving haemodialysis [6]. The continuously growing population of patients receiving haemodialysis as a result of the increasing prevalence of the aforementioned lifestyle diseases or social and demographic factors associated with them has an undoubted impact on this fact. Attempts to find new applications for OACs in this group of patients are, however, not less important. Although they are based on a number of prospective, randomised studies in the general population, there are no such studies in the group of patients receiving haemodialysis [7]. Attempts to apply the results of studies carried out in the general population, from which patients with end-stage renal failure are usually excluded to begin with, to patients receiving haemodialysis are not only unjustified but sometimes have downright negative influence on the effectiveness of treatment and patients' safety.

## 2. Chronic Kidney Disease and Haemostasis Disorders

As renal failure progresses, increasingly significant disturbances occur in the process of blood coagulation. At the initial stages of CKD, mostly as a result of disorders of the plasma coagulation system and fibrinolysis (e.g., decreased levels of protein C and antithrombin III, elevated concentrations of fibrinogen, von Willebrand factor, factor VIII, elevated concentration of plasminogen activator inhibitor-1 (PAI-1), decreased concentration of tissue plasminogen activator (t-PA)), prothrombotic processes, clinically expressed as hypercoagulation, dominate [8, 9]. As glomerular filtration rate (GFR) decreases and renal failure progresses, uraemic bleeding diathesis, characteristic of end-stage renal failure and patients during dialysis therapy, worsens. At the end stages of CKD, the accumulating uraemic toxins, both low-molecular-weight (e.g., urea, phenol and guanidinosuccinic acid) and medium-molecular-weight ones (e.g., RGD polypeptides), affect mostly platelet function, inhibiting their adhesion and aggregation and releasing platelet factors, such as serotonin or thromboxane A_2_ [10, 11]. These phenomena mostly lead to platelet haemostasis disorders. Uraemic toxins and proinflammatory cytokines, frequently co-occurring lipid disorders or arterial hypertension damage endothelium and in this way disrupt also vascular haemostasis. As a result of their stimulation, endothelial cells produce large amounts of prostacyclin (PGI_2_) and nitric oxide (NO) [12]. The former is a strong inhibitor of platelet aggregation and the latter of platelet adhesion. Their elevated concentrations, therefore, increase the existing haemorrhagic diathesis.

A clear bleeding tendency does not exclude, of course, a simultaneous state of increased prothrombotic susceptibility even in the same patient. The peculiar competition between these two antagonistic systems is presented in Table 1, which includes factors that occur as renal failure progresses, predisposing the patient to bleeding events and, on the other hand, to the formation of thrombi [13]. The factors conducive to bleeding mentioned in the table, as we can see, mostly disrupt platelet haemostasis; they disrupt vascular haemostasis to a lesser extent, mainly those that do not take part in the process of coagulation directly. On the other hand, prothrombotic factors include, apart from those affecting plasma haemostasis, first and foremost, the state of endothelium and accelerated atherosclerotic processes inextricably linked with end-stage renal failure [14]. Most frequently, this dynamic equilibrium is slightly shifted towards haemorrhagic diathesis, which is suggested by the clinical characteristics of the haemodialysed patient; the results of his or her additional tests usually increased bleeding time with usually normal APTT and INR. This state of equilibrium is, however, extremely unstable and the slightest additional factor affecting the processes of blood coagulation/fibrinolysis shifts it to either of the sides.

## 3. Chronic Kidney Disease and Oral Anticoagulant Treatment

The applied medications are a factor which frequently and significantly disturbs the above-described equilibrium. Vitamin K antagonists are still the most commonly used oral anticoagulant medications (incidentally, the erroneous name “antagonists” has been adopted, though they do not have antagonist effects on vitamin K, only inhibitory ones, so, to be precise, they are its inhibitors). The first medicine from this group was dicoumarol, isolated by Karl Link at the University of Wisconsin in 1941 [15]. However, they really started to be commonly used in 1950, when a more effective and bioavailable medicine was introduced—warfarin [16]. Their mechanism of action consists in inhibiting the activity of the vitamin K reductase complex, which makes the carboxylation of the residues of glutamic acid in the N-terminal fragments of different proteins impossible. This way, the activity of four key factors of the blood coagulation system, factors II, VII, IX, X, is inhibited [17]. It is a strong anticoagulant activity, because key factors both to the auxiliary endogenous pathway and, first and foremost, to the exogenous pathway, fundamental to the coagulation system, are inhibited. At therapeutic doses, the inhibition of the aforementioned coagulation factors should range from 30 to 50% [18]. This very narrow therapeutic window can, on the one hand, result in the ineffectiveness of treatment, and, on the other, in frequent serious adverse reactions, mostly severe bleeding events. OACs in the blood strongly bind to albumins, but the active free fraction fluctuates in a fairly wide range (from 0.5 to 3%) [19]. Elimination occurs via hepatic metabolism by various cytochromes for each of the isomers of the active ingredient of the medicine; its inactive metabolites are excreted by the kidneys [20]. The half-life is from 18 to 70 hours [21]. It would seem that the hepatic metabolism of coumarin derivatives justifies the use of these medications in patients with end-stage renal failure and receiving haemodialysis following the same rules and at the same doses as in the general population. However, the above-described pharmacodynamic and pharmacokinetic properties make treatment with OACs in patients receiving haemodialysis extremely difficult. The therapeutic window becomes even narrower, the concentrations of the free fraction of the medicine fluctuate even more, depending on changes in peridialytic volaemia, and the amount of fraction bound to albumins decreases as a result of malnutrition or patient cachexia [22]. Moreover, there are a number of known nutritional limitations associated with using OACs, restrictions in the treatment of elderly individuals (slower hepatic metabolism) or their numerous interactions with other medicines (especially antiplatelet ones) [23]. In the conditions of advanced CKD, the impact of all these factors is much greater [24].

Maruyama et al. [25], evaluating INR and albumin values before and after HD procedures, demonstrated a significant decrease in the value of INR following HD in comparison with its levels determined prior to HD, while albumin concentrations increased significantly following the procedure in relation to values prior to dialysis. What is more, the authors also showed a significant, negative correlation between both of the above-described parameters during haemodialysis. Abe et al. [26], apart from similar changes regarding INR and proteins during HD, demonstrated a significant rise in warfarin concentrations post-HD versus pre-HD, both with respect to the more potent S-isomer, and the weaker R-isomer. Interestingly, multifactorial analysis revealed the strongest links between peridialytic values of INR and albumin concentrations. The ability of blood proteins, especially albumin, to bind warfarin is very strong, and therefore changes in their concentration during HD result in significant changes in the concentrations of the active ingredient of the medicine, INR, and, consequently, treatment complications. In a retrospective study of 142 haemodialysed patients who received acenocoumarol for typical indications at a dose of 1 mg a day for a year, Gompou et al. [27] demonstrated that 30% of the INR values determined at this time were below target values, 37% were in the therapeutic range, and nearly 33% exceeded therapeutic values. Admittedly, there is no information regarding the severe complications of such treatment in the report, but the fact that OACs were overdosed in 1/3 of the cases seems alarming.

So far, among patients with end-stage renal failure and patients receiving haemodialysis, no prospective, randomised studies evaluating the effectiveness and safety of OACs have been conducted. In spite of that, in this group of patients, these medications are attempted to be used according to their typical indications, taking, however, special precautions in using them due to the considerably increased risk of adverse reactions [28]. Indeed, the literature includes a number of reports of such reactions, including very severe and fatal ones [29–31]. It is also interesting to read the official contraindications for the use of OACs approved in registration documents by regulatory bodies authorised to do that [32] (e.g., the FDA) (Table 2). On the list presented in Table 2, almost every other item (e.g., 1, 3, 5, 7, and the last one) refers to patients with end-stage renal failure, including patients receiving haemodialysis. Admittedly, chronic renal failure is not explicitly listed, but the items enumerated above refer directly to it. In any case, most of them overlap with factors predisposing to bleeding events in patients with end-stage renal failure and receiving haemodialysis as described earlier (Table 1). As we can see, platelet function disorders, other thrombocytopathies, the risk of gastrointestinal bleeding, urinary tract bleeding, falls, or lack of adequate collaboration in this specific treatment are especially significant in this respect. After all, it is a picture of patients that we see in nephrology units or at dialysis centres on a daily basis. Is, therefore, OAC treatment according to its typical indications in patients with end-stage CKD or in those receiving haemodialysis an off-label use? an experimental one? and therefore, is it justified? these questions remain open.

## 4. OACs in Patients with End-Stage Renal Disease in Clinical Practice

Attempts to use OACs in patients at the end stages of CKD and receiving haemodialysis encounter a number of difficulties. Practically all prospective, controlled, randomised studies, which evaluated the effectiveness and safety of OACs in the general population, carried out so far excluded patients with creatinine clearance <30 mL/min. Applying the results of these studies to the population with advanced renal failure should not therefore be automatic, because their simple use not only may prove to be ineffective in this group of patients, but, what is worse, may be dangerous to them as well.

### 4.1. OACs in the Treatment of Venous Thromboembolism in Patients with CKD

The prevalence of pulmonary embolism in the population of patients receiving haemodialysis is several times higher than its prevalence in the general population. The annual incidence of pulmonary embolism in this group of patients is approximately 15/100,000 versus 25/100,000 patients in the general population [33]. However, there are no evidence-based studies evaluating the use of OACs in this disease and in this population. The remaining option, therefore, is beginning treatment with unfractionated heparin, usually on an inpatient basis, then changing it over the next several days and continuing the OAC treatment while monitoring INR, which is the treatment accepted generally for the population without renal failure [34]. Due to the lack of relevant research, the length of therapy depending on the aetiology of the disease, the individual risk of thrombosis/bleeding events for a given patient and often the decision of the patient him- or herself regarding the applied OAC treatment remain to be evaluated. In the case of absolute contraindications for OACs, implantation of a filter in the inferior vena cava could be an alternative therapy, but there are no such studies for patients receiving haemodialysis in the available literature, and they are inconclusive even in the general population [35].

### 4.2. OACs in Preventing Thrombosis of Vascular Access for Haemodialysis

It is estimated that the causes of almost 1/3 of cases requiring hospitalisation among patients receiving haemodialysis are associated with vascular access failure [40]. The likelihood of the occurrence of vascular access failure due to thrombosis in over a year-long observation of patients receiving haemodialysis is approximately 15% in the case of autologous arteriovenous fistulae and two times higher in the case of vascular grafts [41]. It would therefore seem that OACs can be an effective treatment option in reducing this risk.

Several such prospective, controlled studies (some of which were even randomised) have been conducted, though on small groups of patients. Their results, however, proved to not be very encouraging. In a group of 75 patients receiving haemodialysis, OAC treatment, whose therapeutic target was INR 1.5–2.0, was found to be only slightly more effective in the prevention of thrombotic complications in comparison with the control group, in which OAC treatment was not initiated (66% versus 57%), but the recorded difference was not statistically significant [42]. What is more, in spite of the unchanging study protocol, the target INR values were achieved in only a half of the subjects. While serious bleeding events were not recorded, the target INR range was not high. In another randomised study on a group of 144 patients receiving haemodialysis, Colì et al. [43] used OACs with a little higher therapeutic range of INR (1.8–2.5). Statistically significant differences between the study group and the control group not treated with OACs were observed only after taking the time at which OAC treatment was initiated into consideration. In the group in which this treatment was initiated in the first 24 hours after the implantation of a vascular catheter, vascular complications were found in a little more than 10% of patients, in comparison with patients who received OACs after their first thrombotic event, where the proportion of complications was a little over 50%. In another study, carried out on a group of 63 patients receiving haemodialysis, the use of OACs, whose therapeutic aim was INR of 2.0-3.0, was proved to be significantly more effective in preventing thrombosis than in the group of patients not receiving such treatment [44]. However, when the results of the group treated with OACs were compared with the group in which acetylsalicylic acid at a dose of 325 mg/day was used, differences in the effectiveness regarding vascular access thrombosis were not observed. Serious bleeding events, which significantly more frequently occurred in the group receiving OACs, were the only significant difference between these groups.

How effective is, therefore, the application of OACs in this group of patients? As the few, not very reliable studies suggest, it, admittedly, increases with the increase in the target INR but at the price of significant adverse reactions in the form of serious bleeding events. Moreover, in the last of the cited studies, which confirmed such effectiveness, similar effectiveness was recorded with the use of acetylsalicylic acid, without severe complications. The results of the above studies and the opinions of experts in the area do not allow, therefore, a routine use of OACs due to the discussed indications [45]. This is because a target, safe INR range for this group of patients has not been established, the effectiveness of the medications is doubtful and the risk for the patient is high; furthermore, there are alternative methods that are no less effective and, as it appears, are safer [44].

### 4.3. OACs in the Prevention of Thromboembolic Complications Associated with Atrial Fibrillation

Atrial fibrillation (AF) is the most common cardiac dysrhythmia. It is estimated that it affects several percent of the population over the age of 65 and the proportion increases with age [46]. However, in the group of patients with end-stage renal failure, this proportion is much higher and reaches, according to various studies, from 10 to as high as 30% [47]. The annual mortality rate among these patients is approximately 25% in the general population and almost 30% in patients receiving haemodialysis [48]. The prevalence of stroke in patients with atrial fibrillation without renal failure is, on the other hand, close to that in patients receiving haemodialysis (17% versus 15%, resp.) [49, 50]. Similarly, studies evaluating the risk of stroke among patients receiving haemodialysis depending on the presence of AF or sinus rhythm are inconclusive. Some of them show a several-fold increase in the incidence of stroke in the group with AF, while others do not record such a correlation [50, 51]. These two factors, among other things, are probably responsible for the inconclusive results of studies evaluating the effectiveness and safety of OAC treatment due to AF in the group of patients with end-stage renal failure and patients receiving haemodialysis. The confusion in the literature regarding the plausibility and significance of the discussed issue is increased, however, mostly by the fact that there are simply no prospective, controlled, randomised studies in this group.

First, let us look at the impact of the use of OACs in this group of patients on the hard end-point, which is patient mortality (Table 3). Knoll et al. [36], carrying out a study on a group of 235 haemodialysed patients with AF receiving OACs, recorded a slightly lower mortality rate than that which characterised the control group, not receiving OACs, although not significant. Chan et al. [37], in a retrospective study conducted on more than 1,600 patients with AF receiving haemodialysis, showed that the use of warfarin in the prevention of thromboembolic events is not associated with all-cause mortality or an increase in the number of hospitalisations. In turn, Wizemann et al. [38] observed a clear increase in mortality rate in patients receiving warfarin for the same reasons as in haemodialysed ones, which was especially apparent in the older population (>75 years old). Another study confirmed the negative impact of OACs used for the same indications on the mortality rate in patients with end-stage renal failure in comparison with the control group, which did not receive such treatment [39].

Data regarding effectiveness in decreasing the risk of stroke in patients with AF receiving haemodialysis are also inconclusive. In one of the latest of such studies, Olesen et al. [52], in a group of more than 900 patients in whom warfarin was used in fewer than 20% of cases, observed for a period of 11 years and found a statistically significant decrease in the risk of the occurrence of stroke or another systemic thromboembolic event. However, in several other studies, such an effect was not observed; what is more, the effect of using OACs was decidedly negative. In the study cited above, Chan et al. [37] recorded a two times higher risk of stroke in the group treated with warfarin than in the control group not receiving OACs. Furthermore, there was an increase in the risk of both haemorrhagic and ischaemic stroke (HR 2.2 and 1.8, resp.). Similar results were presented by Wizemann et al. [38] in a group of 3,245 patients participating in DOPPS I and DOPPS II (Dialysis Outcomes and Practice Patterns Study). In this study, the patients' age was the factor differentiating the risk. In the oldest group (>75 years old), warfarin treatment was associated with a significant, more than twofold, increase in the risk of stroke; in younger groups (65–75 and <65), the increase in the risk of stroke was evident but statistically insignificant. In another study, which covered 2,313 haemodialysed patients with newly diagnosed AF and treated with warfarin for the first time, Winkelmayer et al. [53] observed an over twofold increase in the risk of haemorrhagic stroke; such a relationship was not found in the case of ischaemic stroke.

The risk of adverse reactions, including severe bleeding events, during OAC treatment in patients receiving haemodialysis rises considerably. Previous studies, in which OACs were used for different indications, had already reported that [54]. In the case of the treatment of AF, the situation looks similar; most reports suggest a several-fold increase in this risk. The results of the study by Chan et al. [39] suggest a significant, almost threefold increase in the risk of severe bleeding events during OAC treatment in haemodialysed patients due to AF. In the same study, clopidogrel was associated with a more than 2.5-fold (higher) risk of significant haemorrhagic complications; what is interesting is that the use of acetylsalicylic acid also increased this risk, but not significantly. Holden et al. [55], in turn, observed an increase in such risk, which was more than threefold in the case of warfarin and over fourfold in the case of acetylsalicylic acid; the use of these medications in combination was associated with more than a sixfold increase in this risk. Other studies also suggested a significant risk of severe bleeding events associated with the use of OACs in the prevention of thromboembolic complications due to AF [52, 56].

Is, therefore, OAC treatment for the above indications in patients receiving haemodialysis effective and safe? Do the benefits of using OACs outweigh the risk of severe bleeding events in patients with end-stage renal failure?

All of the above study results should be treated cautiously. As we have mentioned, there are no large-scale, controlled, prospective, randomised studies here. Only on the basis of such studies would it be possible to present guidelines or recommendations regarding OAC treatment in this group of patients. Most of them are studies conducted on small groups, observational, retrospective groups; only a few of them were prospective, but not randomised. It is also difficult to perform a reliable meta-analysis, because the individual studies sometimes differ in their design, specific assumptions, methods of collecting data, or methods of analysis [37, 38, 53]. Decisions regarding the initiation of OAC treatment and the time when it occurred depended mostly on the patient's clinical data and the physician's experience. Some studies also excluded patients taking OACs or patients with AF that had developed before renal failure. All these circumstances make it necessary to evaluate these studies cautiously.

Moreover, let us look at two epidemiological issues [49–51]. The prevalence of stroke in patients with atrial fibrillation without renal failure, in comparison with that in patients receiving haemodialysis, turned out to be comparable in the above-cited studies. However, the evaluation of the risk of stroke in patients receiving haemodialysis depending on the presence of AF or sinus rhythm proved to be inconclusive. If, therefore, there is no such risk, what role are OACs supposed to play? Perhaps a significant portion of the causes of stroke in the group with end-stage renal failure are vascular ones and atherosclerosis, considerably accelerated in this group of patients? In this situation, OACs would not change such risk significantly.

In turn, the increase in the risk of death and the increase in the incidence of stroke in haemodialysed patients treated with OACs may be explained by two things. The first one is, of course, inappropriate management of anticoagulation, or rather the time during which the patient remains in the desired INR range. After all, its deviation towards higher or lower values is associated with an increase in morbidity or adverse reactions. On a daily basis, in nephrology units or dialysis centres, we can see how difficult it is to keep INR in such a therapeutic range in patients receiving haemodialysis. It is associated with the fact that chronic renal failure, and haemodialysis therapy to an even larger extent, are a significant and independent factors that may decrease the period in which the desired value of INR is maintained [57]. Anyway, the above-cited data regarding the increase in the risk of bleeding events during OAC treatment in patients receiving haemodialysis most likely result from the same cause. The described impact of vitamin K antagonists on the processes of vascular calcification and calciphylaxis is another noteworthy, potential factor. As a number of studies, both experimental and clinical, show, vitamin K inhibition, for example, by OACs, inhibits the activity of matrix Gla protein (MGP), an important protein, playing a preventive role in vascular calcification [58, 59]. OACs can therefore directly interfere and accelerate the process of vascular calcification, already increased in end-stage renal failure. Is it a causative factor, significantly raising the incidence of ischaemic stroke and mortality among haemodialysed patients receiving OACs? This question must be answered by future studies.

Patients with end-stage renal failure, including, above all, those who receive haemodialysis, are a group of patients with exceptionally many concomitant diseases [60]. Considerably accelerated atherosclerotic processes, vascular calcification, chronic inflammation, lipid disorders, and malnutrition are inherent characteristics of this population of patients. Can OACs have an impact on the risk of stroke in haemodialysed patients with AF in these conditions and with the existing vascular changes also within the central nervous system?—this Question remains open. However, the results of the cited studies do not allow us to use OACs routinely for these indications in this group of patients.

## 5. Conclusions

The population of patients with CKD, including patients receiving haemodialysis, keeps growing all over the world. However, OAC treatment in this group of patients, irrespective of its indications, causes a great deal of controversy. This is because a number of pro- and anticoagulant factors play an important role in end-stage renal failure due to the nature of the disease itself. In these conditions, the pharmacokinetic and, especially, pharmacodynamic properties of the OACs used change as well. All these factors make proper anticoagulation in these patients more difficult, and, most importantly, they decrease the time during which patients remain in the therapeutic range of INR. In the case of the treatment of venous thromboembolism, the only remaining option is OAC treatment according to regimens used for the general population, as there are no relevant studies concerning the group of patients receiving haemodialysis. Prevention of HD vascular access thrombosis with the use of OACs, according to the presented studies and experts' opinions, is not very effective and can additionally be very dangerous. However, OAC treatment in haemodialysed patients with AF in order to prevent thromboembolic events, according to some authors, is associated with an increase in the incidence of stroke and mortality.

Is the provocative question included in the title justified? Doubts should be dispelled by prospective, controlled, randomised studies; at the moment, there is no justification for routine use of OACs in the above-mentioned indications. In selected cases of OAC treatment in this group of patients, it is absolutely necessary to control and monitor the applied treatment thoroughly. According to the authors and the opinions already partially reported in the literature [45], indications for the use of OACs in patients with end-stage renal failure, including patients receiving haemodialysis, should be limited to those included in Table 4.

## Figures and Tables

**Table 1:** Factors conducive to coagulation disorders in patients with end-stage renal disease and receiving haemodialysis [8–13].

Factors conducive to bleeding events	Factors conducive to thrombosis
*Directly *	
Platelet adhesion disorders—decreased activity of von Willebrand factor and receptor GPIb, increased release of PGI_2_, NO Platelet aggregation disorders—decreased activity of GPIIb/IIIa receptor, impaired binding of fibrinogen to platelets Platelet secretion disorders—decreased production of thromboxane A_2_, serotonin and ADP, decreased release of *β*-thromboglobulin, and impaired Ca^++^ mobilization Reduced number and volume of platelets	Accelerated atherosclerotic processes, damaged endothelium Defective GPIb expression on the surface of platelets Disorders of protein C metabolism, decreased concentration of protein C and antithrombin III Elevated concentrations of plasminogen activator inhibitor-1 (PAI-1)

*Indirectly *	
Anaemia—altered rheological features of blood, impaired platelet aggregation Uraemic toxins—for example, PTH Medications—antiplatelet, anticoagulant, cephalosporins, antitubercular, inhibitors of lipid absorption, NSAIDs Concomitant diseases—for example, affecting the gastrointestinal tract Invasive procedures—cannulations, biopsies, vascular access	

GP: glycoprotein, PGI_2_: prostacyclin, NO: nitric oxide, ADP: adenosine diphosphate, Ca: calcium ions, PTH: parathormone, NSAIDs: non-steroidal anti-inflammatory drugs.

**Table 2:** Contraindications for the use of oral anticoagulants [32].

(i) **Increased risk of bleeding events—platelet function disorders, thrombocytopenia, von Willebrand disease, and haemophilia**	
(ii) Recent intracranial bleeding. Conditions predisposing to intracranial bleeding—cerebral artery aneurysms	
(iii) **Conditions predisposing to gastrointestinal, urinary, and respiratory tract bleeding**	
(iv) Surgical procedures within the central nervous system or the eye	
(v) **Increased risk of frequent falls—caused by a neurological or another condition**	
(vi) Severe liver failure, cirrhosis	
(vii) **Untreated or poorly controlled arterial hypertension**	
(viii) Pregnancy	
(ix) Infective endocarditis or pericardial effusion	
(x) Hypersensitivity to the active substance or any of the excipients	
(xi) **Dementia, psychoses, alcoholism, and other conditions in which compliance may not be satisfactory and when anticoagulant treatment cannot be safely administered**	

End-stage renal-disease-associated items are bold.

**Table 3:** Mortality risk in haemodialysis patients with atrial fibrillation treated with warfarin.

Study	Population (*n*)	Period (y)	Mortality (HR)
Knoll et al. [36]	*n*—235 pts	3 years	HR—0.80 (95% CI 0.28–2.29, *P* < 0.67)

Chan et al. [37]	*n*—1671 pts	1 year	HR—1.10 (0.94–1.30)

Wizemann et al. [38]	*n*—17513 pts (AF—12.5%)	7 years	All ages: HR—1.16 (95% CI 1.08–1.25, *P* < 0.001)
			Age < 65: HR—1.29 (95% CI 0.45–3.68, *P* < 0.63)
			Age 65–75: HR—1.35 (95% CI 0.69–2.63, *P* < 0.39)
			Age > 75: HR—2.17 (95% CI 1.04–4.53, *P* < 0.04)

Chan et al. [39]	*n*—41425 pts	11 years	
	Warfarin—8.3%		HR—1.73 (95% CI 1.62–1.85)
	Clopidogrel—10%		HR—1.50 (95% CI 1.39–1.62)
	Acetylsalicylic acid—30.4%		HR—1.17 (95% CI 1.12–1.22)
	Acetylsalicylic acid and warfarin—8%		HR—1.11 (95% CI 1.03–1.86)

HR: hazard ratio, CI: confidence interval, *n*: number of patients.

**Table 4:** Potential indications for oral anticoagulants in patients with end-stage renal disease and receiving haemodialysis.

(i) Status after prosthetic heart valve implantation	
(ii) Antiphospholipid syndrome	
(iii) Secondary prevention of severe thromboembolic events (for example, pulmonary embolism)	
(iv) Atrial fibrillation with a high risk of stroke	
